# A prenatal acoustic signal of heat affects thermoregulation capacities at adulthood in an arid-adapted bird

**DOI:** 10.1038/s41598-022-09761-1

**Published:** 2022-04-07

**Authors:** Anaïs Pessato, Andrew E. McKechnie, Mylene M. Mariette

**Affiliations:** 1 Centre for Integrative Ecology, School of Life & Environmental Sciences, Deakin University, Geelong, 3216 Australia; 2 South African Research Chair in Conservation Physiology, South African National Biodiversity Institute, Pretoria, 0001 South Africa; 3 DSI-NRF Centre of Excellence at the FitzPatrick Institute, Department of Zoology and Entomology, University of Pretoria, Pretoria, 0001 South Africa; 4 Doñana Biological Station EBD-CSIC, 41092 Seville, Spain

**Keywords:** Behavioural ecology, Ecophysiology

## Abstract

Understanding animal physiological adaptations for tolerating heat, and the causes of inter-individual variation, is key for predicting climate change impacts on biodiversity. Recently, a novel mechanism for transgenerational heat adaptation was identified in a desert-adapted bird, where parents acoustically signal hot conditions to embryos. Prenatal exposure to “heat-calls” adaptively alters zebra finch development and their thermal preferences in adulthood, suggesting a long-term shift towards a heat-adapted phenotype. However, whether such acoustic experience improves long-term thermoregulatory capacities is unknown. We measured metabolic rate (MR), evaporative water loss (EWL) and body temperature in adults exposed to a stepped profile of progressively higher air temperatures (T_a_) between 27 and 44 °C. Remarkably, prenatal acoustic experience affected heat tolerance at adulthood, with heat-call exposed individuals more likely to reach the highest T_a_ in morning trials. This was despite MR and EWL reaching higher levels at the highest T_a_ in heat-call individuals, partly driven by a stronger metabolic effect of moderate activity. At lower T_a_, however, heat-call exposed individuals had greater relative water economy, as expected. They also better recovered mass lost during morning trials. We therefore provide the first evidence that prenatal acoustic signals have long-term consequences for heat tolerance and physiological adaptation to heat.

## Introduction

Life-history traits, reproductive success and survival are strongly tied to animals’ capacity to regulate their body temperature across a wide range of environmental conditions^1,2^. Recent evidence demonstrates that heat dissipation capacities in endotherms not only limit their survival under extreme heat^3^ but also their reproductive output during sustained hot conditions^4,5^. These effects are expected to be particularly pronounced in diurnal animals, and those inhabiting hot deserts, exposed to extreme heat and solar radiation^6^. As the severity of climate change—and heatwaves—intensifies^7^, it is crucial to understand species’ potential to adapt to elevated temperatures. Recent models for desert avian communities predict major population declines under forecasted climate change, through increased risks of lethal dehydration, hyperthermia, decline in body mass, and reproductive failure^8–10^.

Physiological mechanisms for endotherm thermoregulation at high environmental temperatures involve evaporative heat dissipation, primarily via respiratory (e.g. panting) or cutaneous (e.g. sweating in some mammals) pathways^11^. Establishing how selection can act on organisms’ thermoregulatory capacities requires identifying the sources of variation in relevant traits. Increasing evidence demonstrates that thermoregulatory capacities and strategies vary between species^12,13^ and populations^14,15^, and importantly, are repeatable within individuals^16,17^. Nonetheless, individual thermoregulation capacities are also known to vary in response to short-term thermal acclimation^18,19^. Although such short-term phenotypic flexibility likely contributes to individual survival, it may, by lowering the strength of selection, reduce opportunities for genetic adaptation under climate change^20^. By contrast, other forms of plasticity, namely developmental plasticity, may benefit population persistence, by generating additional inter-individual variation in phenotypes^21^.

Early-life conditions are well known to profoundly affect individual development and traits, and potentially allow adaptive programming of individual phenotypes to particular environments^22,23^. In ectotherms, beyond the noticeable temperature-dependent sex determination in some reptiles^24^, developmental temperature is also known to influence individual thermal performance curves. For example, in tropical reef fish, high developmental temperatures (+ 1.5 and 3.0 °C above present-day temperature), but also parental exposure to these high temperatures, improved offspring metabolic performance (e.g. reduced resting metabolic rate, increased factorial aerobic scope) in warmer water^25^. Comparable studies of endotherms are far fewer and mainly limited to effects at early life-stages^26^. In birds, exposure to high air temperatures either pre- or postnatally increases young individuals´ capacity to maintain lower body temperature in hot conditions^27–29^, including through improved evaporative cooling efficiency in the rock pigeon (*Columba livia*)^30^. Surprisingly, adaptive developmental programming for high temperatures also occurs via prenatal acoustic communication^31^.

Prenatal acoustic communication occurs in a diverse range of avian taxa^32^. Whilst its role in hatching synchronisation or incubation solicitation at sub-optimal temperatures has long been known, its function for developmental programming was only recently proposed^31,33^. In the desert-adapted zebra finch (*Taeniopygia guttata*), parents emit a special “heat-call”, only at high air temperatures^16,31,34^. Heat-calls are produced through an enhanced form of panting or “vocal panting”, which increases the heat tolerance of the emitter, at the cost of higher water loss^16^. Heat-call utterance is initiated at an individual-specific air temperature threshold (≥ 35 °C^16^), and is higher in late incubation than in other breeding stages or in non-breeding inviduals^31,34,35^. Remarkably, embryonic exposure to these calls adaptively reduces nestling growth at high temperatures, which increases their reproductive success as adults^31^. In addition, individual thermal preferences at adulthood shift towards hotter breeding nests^31^. These findings suggest that prenatal exposure to heat-calls shaped individual thermal physiology towards a heat-adapted phenotype that persisted into adulthood, but this remains to be tested empirically. Furthermore, in yellow-legged gulls (*Larus michahellis*), prenatal acoustic experience was recently found to affect several physiological traits such as telomere length and basal corticosterone level^36,37^, which suggests a direct impact of prenatal sounds on physiology^33^.

Heat-directed phenotypes are observed in individuals acclimated to high temperatures or from hot-climate populations. Such phenotypes show greater tolerance to high air temperature extremes^19,38^, and more efficient evaporative cooling in hot conditions, characterised by shallower increases in resting metabolic rate and/or evaportive water loss^38,39^. Such differences are expected to be most detectable in challenging conditions, such as when air temperature exceeds typical body temperature, or at the time of day when evaporative cooling demands peak (e.g. morning^40,41^). In addition, at mild T_a_, when evaporative cooling is not needed, heat-adapted individuals may be more efficient at conserving water, notably through changes in skin ultrastructure that reduce cutaneous water loss^18,42^. In ectotherms, heat-directed phenotypes induced by early thermal environments persited into adulthood in some studies^43,44^ but not others^45,46^. In birds, high or low incubation temperatures affect thermal tolerance and metabolism in early life, and potentially basal metabolic rate in adulthood^26,47^. However, we are aware of only one study investigating the long-term effects of early thermal environment on heat dissipation. In Japanese quail (*Coturnix japonica*), the effect of postnatal temperature on bill surface temperature persisted until adulthood, whereas that on bill morphology did not^48^. Overall, therefore, it is still unclear whether early-life conditions have long-term effects on thermoregulation in the heat. Yet, establishing the persistence of heat-directed phenotypes into adulthood is essential to understand inter-individual variation, and therefore the fitness benefits that developmental programming may confer.

We tested the hypothesis that prenatal exposure to heat-calls induces a heat-directed phenotype in adult zebra finches, with long-lasting effects on heat tolerance and thermoregulation. We quantified metabolic rate (MR), evaporative water loss (EWL) and body temperature (T_b_) in 34 male and female wild-derived zebra finches, prenatally exposed to playbacks of either heat-calls (treatment group) or control calls (control group). At adulthood, thermoregulatory responses were measured over a standardised sequence of air temperature (T_a_) stages, increasing from 27 °C up to 44 °C, a value approaching the species’ thermal limit^49^. Evaporative cooling behaviours such as panting in birds and vocal panting (i.e. heat-calling) are more common earlier in the day (at a given environmental temperature)^16,34,40,50^, which suggests higher evaporative cooling demand in the morning. We thus tested individuals in both mornings and afternoons, expecting larger differences between playback groups in the morning. First, we investigated whether prenatal exposure to heat-calls improved adult heat tolerance, as indicated by a lower probability of showing signs of severe heat-stress prompting early termination of the trial. We then tested whether heat-call exposure reduces overall water loss and maximises body-water replenishment, using variation in body mass during and after trials. Second, because activity levels and associated metabolic heat production often vary among conspecific individuals^51^, we assessed playback effects on these two traits across T_a_ stages, for all birds tested. Third, we tested for prenatal playback effects on adult thermoregulation capacity (only in calm birds, as customary^52,53^), at high (above thermoneutrality), then mild, T_a_ (within and below the thermoneutral zone^54^). We predicted that heat-call birds would (i) thermoregulate more efficiently than controls at high T_a_, evident as lower T_b_ and/or greater evaporative cooling capacity (quantified by the ratio of evaporative heat loss to metabolic heat production), and (ii) conserve more water (i.e. lower EWL and greater relative water economy RWE) at mild T_a_, when evaporative cooling is not needed.

## Results

### Heat tolerance and body mass variation

**Figure 1:**
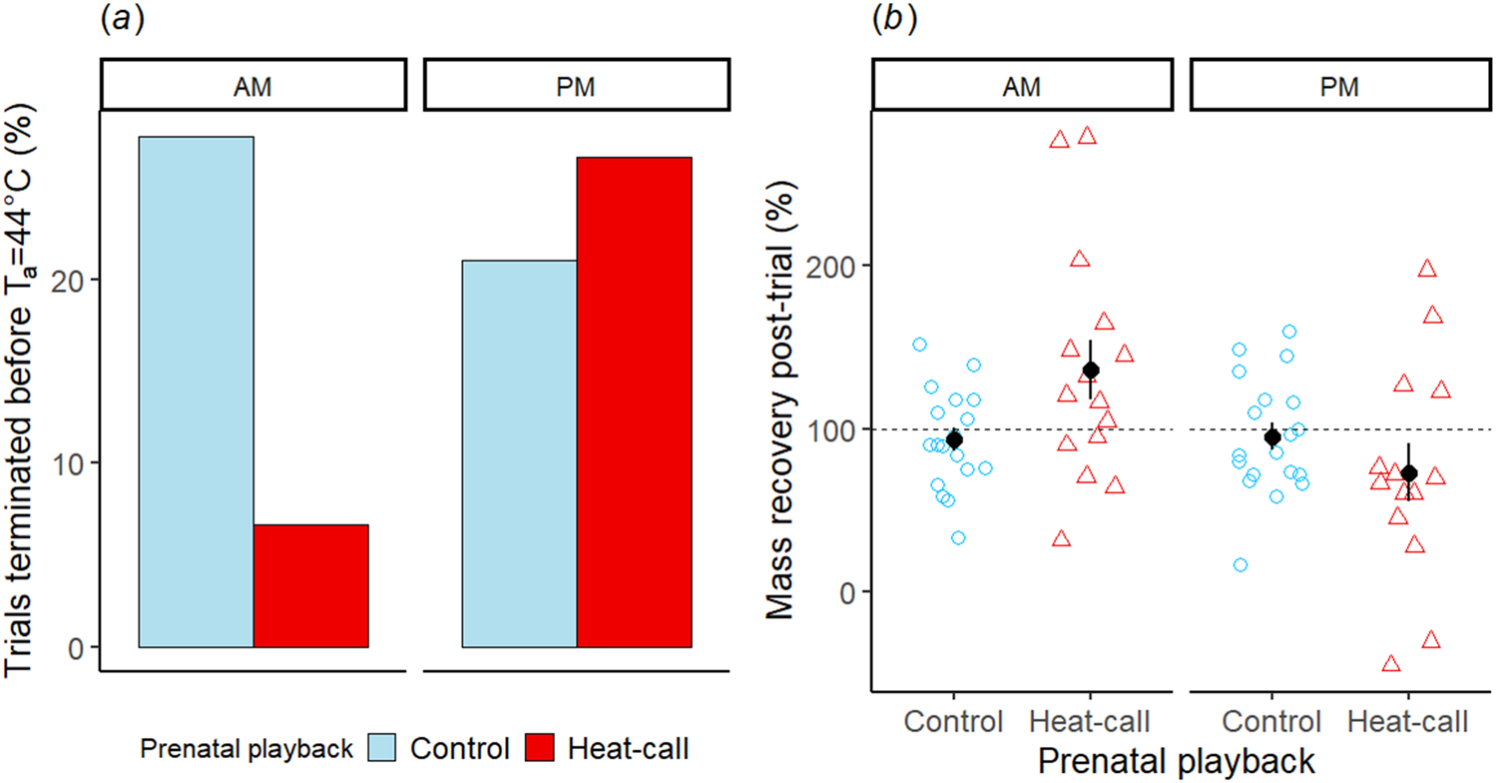
Effects of prenatal playback and time of day (AM or PM) on (**a**) the proportion of trials (n = 67 for 34 birds) terminated before T_a_ = 44 °C on account of birds showing signs of severe heat stress, and on (**b**) post-trial mass recovery (n = 66 for 34 birds). In (**b**), markers and error bars show mean ± SE and the dashed line corresponds to a total recovery of the mass lost during the trial (i.e. 100%). Points are jittered horizontally to facilitate visualisation. Birds had been prenatally exposed to either (i) heat-calls that incubating parents exclusively produce at high temperatures (treatment, red) or (ii) parental contact calls (control, blue).

As predicted, in morning trials, prenatal exposure to heat-calls significantly increased individuals’ likelihood of reaching the highest T_a_ (i.e. T_a_ = 44 °C) during a standardized heat exposure protocol at adulthood (Linear Mixed Model (LMM): playback × time-of-day: est = − 18.718, se = 9.062, p = 0.039): control birds were four times more likely than treatment birds (i.e. 28% versus 7%) to have the morning trial terminated before reaching T_a_ = 44 °C, on account of severe agitation or reaching thermal endpoints (Fig. 1a). However, for individuals that reached T_a_ = 44 °C, the probability of completing the trial (i.e. tolerating T_a_ = 44 °C for 20 min) did not vary with playback (LMM: est = − 3.054, se = 3.755, p = 0.416, Supplementary Table S1).

Prenatal playback and time-of-day also affected body mass fluctuation during and after the trial. Birds lost 0.57 ± 0.02 g (~ 4.7% of their initial mass, mass_init_) on average during a trial, mostly through cumulative evaporative water loss, and regained 0.55 ± 0.004 g (~ 99.2% of that loss) during the hour post-trial, with access to ad libitum food and water. Birds lost more mass during morning than afternoon trials (LMM: est = − 0.061, se = 0.029, p = 0.041), but there was no difference between playback groups (LMM: est = − 0.045, se = 0.062, p = 0.475, Supplementary Table S2). However, for mass recovery, and therefore body water replenishment, treatment birds regained significantly more mass than controls, overall (LMM: est = 42.007, se = 17.436, p = 0.020), but particularly in the morning, when most treatment individuals regained more mass (even up to 2 or 3 times more) than they had lost during the trial (LMM: playback × time-of-day: est = 64.761, se = 18.659, p = 0.002, Fig. 1b, Supplementary Table S2). In the afternoon, however, the pattern tended to be opposite, and most heat-call birds (10/14) did not completely regain their initial mass (Fig. 1b).

### Variation in activity throughout the trial

**Figure 2:**
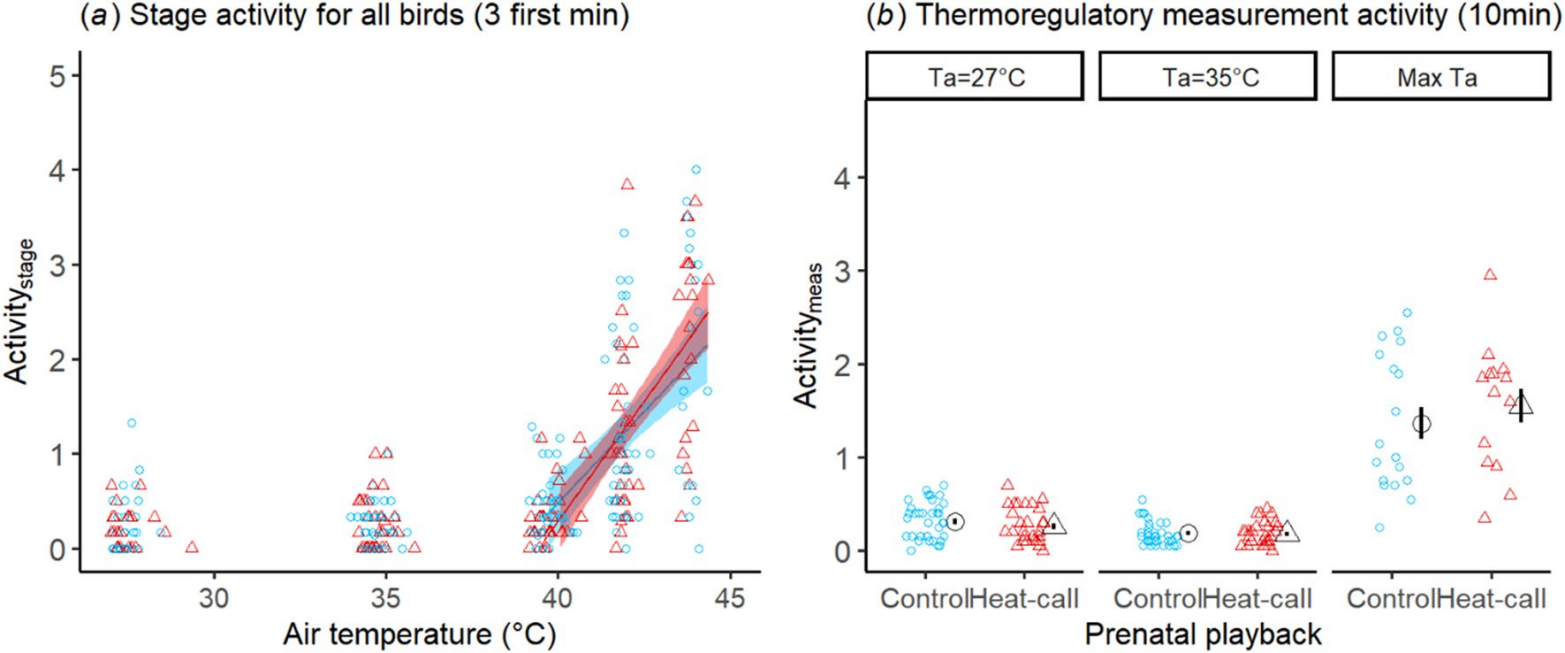
(**a**) Activity over a stepped profile of increasing T_a_ (n = 242 observations), using activity recorded in the first 3 min of stable T_a_ per stage (activity_stage_), for all birds tested (unless trial terminated within < 3 min of T_a_ stage start n = 12). Regression lines display significant relationship from LMM above the inflection point (T_a_ = 39.6 ± 0.5 °C), split by prenatal playback: birds had been prenatally exposed to either heat-calls (red triangles) or control calls (blue dots). (**b**) Activity in the 10 min prior and during measurement (activity_meas_), for calm birds from which we obtained thermoregulatory values, at T_a_ = 27, 35 °C (n = 67) and at the max T_a_ reached (n = 32). Markers and error bars show mean ± SE.

Activity remained low (activity_stage_ < 1.5; i.e. sleeping, sitting or stepping once) in the first three T_a_ stages, but then increased at T_a_ = 42 °C and 44 °C (Fig. 2), when individuals were approaching the species' thermal limit^49^. When all birds tested were considered, activity_stage_ increased more steeply across the T_a_ gradient in treatment than control birds (LMM: playback × T_a_ est = 0.132, s = 0.056, p = 0.021, Fig. 2a, Supplementary Table S3). However, there was no significant difference between playback groups when each T_a_ stage was considered separately (activity_stage_: p > 0.296, Supplementary Table S3). Likewise, for individuals reaching T_a_ = 44 °C, treatment birds were not more agitated than controls at the end of the T_a_ = 42 °C stage (activity_42-end_; LMM: est = 0.184, se = 0.160, p = 0.260, Supplementary Table S4); and, among calm individuals from which we obtained thermoregulatory data, activity (in the 10 min before and during each thermoregulatory measurement [activity_meas_]), was not higher in treatment birds, at any of the T_a_ stages (p > 0.273, Fig. 2b, Supplementary Table S5).

### Thermoregulatory responses to heat

**Figure 3:**
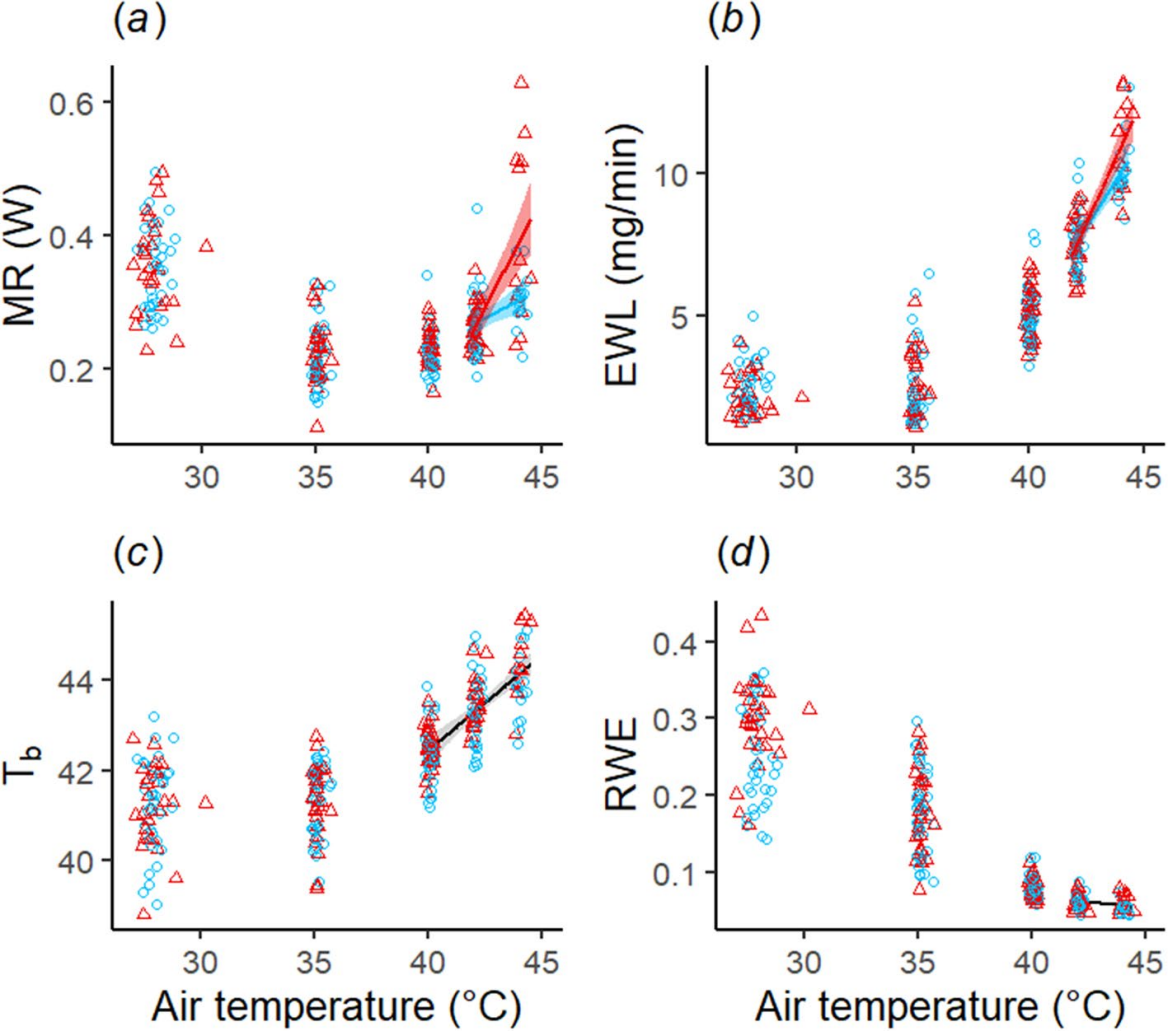
(**a**) Metabolic rate (MR), (**b**) evaporative water loss (EWL), (**c**) body temperature (T_b_), and (**d**) relative water economy (RWE) of calm birds over a stepped profile of increasing T_a_. Segmented regressions identified a significant inflection point at T_a_ = 41.2 ± 0.3 °C, 40.7 ± 0.3 °C, 40.2 ± 0.9 °C, and 40.8 ± 0.5 °C, for RMR, EWL, T_b_, and RWE. Regression lines display significant relationship from LMMs above inflection points, split by prenatal playback when significant: birds had been prenatally exposed to either heat-calls (red triangles) or control calls (blue dots).

In addition to improving heat tolerance, prenatal acoustic experience altered individual thermoregulation above the upper critical limit of thermoneutrality (Fig. 3, Supplementary Table S6). Playback affected how MR and EWL increased with T_a_ (LMMs : playback × T_a_ p = 0.002 and p = 0.044 respectively). However, similarly to activity, the slope of increase was steeper in heat-call than control birds (Fig. 3a,b).

**Figure 4:**
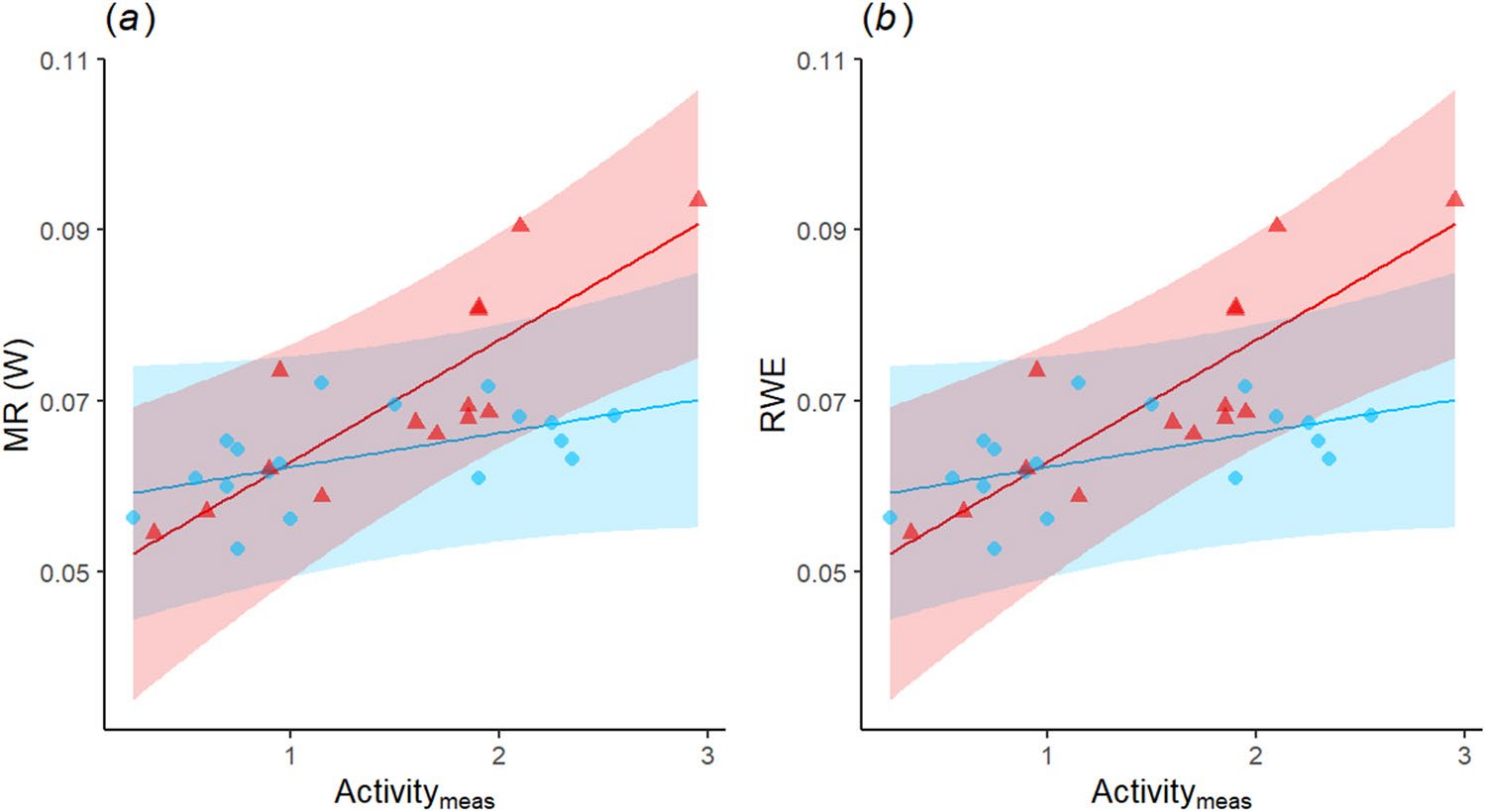
Partial residual plot for the effects of prenatal playbacks and activity on (**a**) metabolic rate (MR) and (**b**) relative water economy (RWE) at Max T_a_ reached (n = 32 observations for 19 birds), for birds prenatally exposed to either heat-calls (red triangles) or contact calls (blue dots). Regression lines and confidence intervals (for significant effects) were plotted using the function *interact_plot* from *interactions* R package.

When considering specifically the most extreme T_a_ each individual had reached (i.e. Max T_a_ = 42 or 44 °C), prenatal playback again affected metabolic rate and water loss, in interaction with activity prior and during physiological measurement (activity_meas_). MR and RWE increased with activity_meas_ (and EHL/MHP correspondingly decreased) in treatment but not control birds, resulting in higher physiological values for treatment birds at a given activity level (Table 1, Fig. 4a,b, Supplementary Tables S7 and 8), even though activity_meas_ itself did not differ (see above). In addition, playback had no significant effect on evaporative water loss or T_b_ at the highest T_a_ (Table 1). There was also no diurnal variation in thermoregulatory values at high T_a_ (Table 1), but T_b_ was higher in females than males (Table 1).

**Table 1:** Outputs of reduced linear mixed-effects models of metabolic rate (MR), evaporative water loss (EWL), relative water economy (RWE) and T_b_ of calm individuals at the maximal T_a_ reached (i.e. T_a_ = 42 °C or 44 °C, n = 32 observations for 19 birds), within the thermoneutral zone (T_a_ = 35 °C, n = 67 for 34 birds) and at mild temperature (T_a_ = 27 °C, n = 67 for 34 birds).

Predictors	Est. ± SE	p	Est. ± SE	p	Est. ± SE	p	Est. ± SE	p
	MR		EWL		RWE^a^		T_b_	
**Max T**_**a**_** (42 °C or 44 °C)**								
Intercept	0.238 ± 0.089	**0.014**	9.631 ± 1.153	** < 0.001**	0.048 ± 0.009	** < 0.001**	44.060 ± 0.469	** < 0.001**
Playback	0.063 ± 0.032	0.062	0.607 ± 0.433	0.183	0.005 ± 0.003	0.157	0.181 ± 0.194	0.367
Mass_init_	0.018 ± 0.016	0.271	0.189 ± 0.212	0.385	0.003 ± 0.002	0.095	− 0.025 ± 0.091	0.786
Time	0.022 ± 0.033	0.509	− 0.123 ± 0.408	0.768	0.004 ± 0.003	0.156	0.189 ± 0.143	0.211
Trial	0.030 ± 0.037	0.434	− 1.110 ± 0.473	**0.029**	0.011 ± 0.003	**0.008**	− 0.233 ± 0.175	0.200
Activity_meas_	0.021 ± 0.021	0.348	0.356 ± 0.243	0.156	0.003 ± 0.002	0.177	0.388 ± 0.095	** < 0.001**
Sex	0.014 ± 0.033	0.680	− 0.214 ± 0.451	0.643	0.001 ± 0.004	0.770	− 0.630 ± 0205	**0.009**
T_a_	0.014 ± 0.051	0.785	2.221 ± 0.688	**0.006**	− 0.013 ± 0.005	**0.037**	0.495 ± 0.303	0.122
Playb.xAct_meas_	0.076 ± 0.035	**0.042**			0.007 ± 0.003	**0.039**		
**T**_**a**_** = 35 °C**								
Intercept	0.218 ± 0.017	** < 0.001**	2.345 ± 0.428	** < 0.001**	0.182 ± 0.018	** < 0.001**	41.080 ± 0.266	** < 0.001**
Playback	0.006 ± 0.015	0.692	0.046 ± 0.374	0.903	− 0.003 ± 0.016	0.849	0.018 ± 0.259	0.944
Mass_init_	0.016 ± 0.007	**0.019**	0.291 ± 0.164	0.081	− 0.003 ± 0.007	0.717	0.148 ± 0.097	0.131
Time	− 0.005 ± 0.007	0.469	− 0.411 ± 0.165	**0.019**	0.022 ± 0.007	**0.004**	0.014 ± 0.081	0.860
Trial	− 0.002 ± 0.007	0.773	− 0.077 ± 0.173	0.661	0.003 ± 0.007	0.662	0.137 ± 0.088	0.128
Activity_meas_	− 0.007 ± 0.005	0.134	− 0.069 ± 0.115	0.552	− 0.002 ± 0.005	0.641	− 0.002 ± 0.058	0.966
Sex	0.012 ± 0.015	0.432	0.800 ± 0.373	**0.041**	− 0.035 ± 0.016	**0.042**	0.011 ± 0.258	0.966
**T**_**a**_** = 27 °C**								
Intercept	0.368 ± 0.023	** < 0.001**	3.131 ± 0.334	** < 0.001**	0.198 ± 0.029	** < 0.001**	41.920 ± 0.370	** < 0.001**
Playback	0.003 ± 0.019	0.872	− 0.472 ± 0.245	0.064	0.048 ± 0.017	**0.010**	0.090 ± 0.299	0.766
Mass_init_	0.019 ± 0.008	**0.019**	0.113 ± 0.110	0.307	< − 0.001 ± 0.008	0.994	0.158 ± 0.125	0.211
Time	− 0.013 ± 0.007	0.084	− 0.237 ± 0.118	0.053	0.019 ± 0.012	0.129	− 0.019 ± 0.119	0.874
Trial	− 0.006 ± 0.010	0.530	− 0.372 ± 0.159	**0.024**	0.038 ± 0.015	**0.017**	− 0.407 ± 0.164	**0.017**
Activity_meas_	0.009 ± 0.006	0.162	0.018 ± 0.096	0.853	0.002 ± 0.009	0.822	0.172 ± 0.101	0.095
Sex	0.002 ± 0.019	0.923	0.403 ± 0.243	0.108	− 0.030 ± 0.017	0.096	− 0.132 ± 0.297	0.659

The reference is the control group for playback, morning for time-of-day (here “Time”), trial 1 for trial, and female for sex. Mass_init_ corresponds to the mass measured before starting the trial. Est. ± SE corresponds to estimate ± standard error. Bold font shows significant effect (p < 0.05).

^a^Patterns for evaporative cooling efficiency (EHL/MHP) were always consistent with those on RWE, with the same predictors having significant effects in the opposite direction (see Supplementary Table S8).

### Thermoregulatory responses at thermoneutrality and below

**Figure 5:**
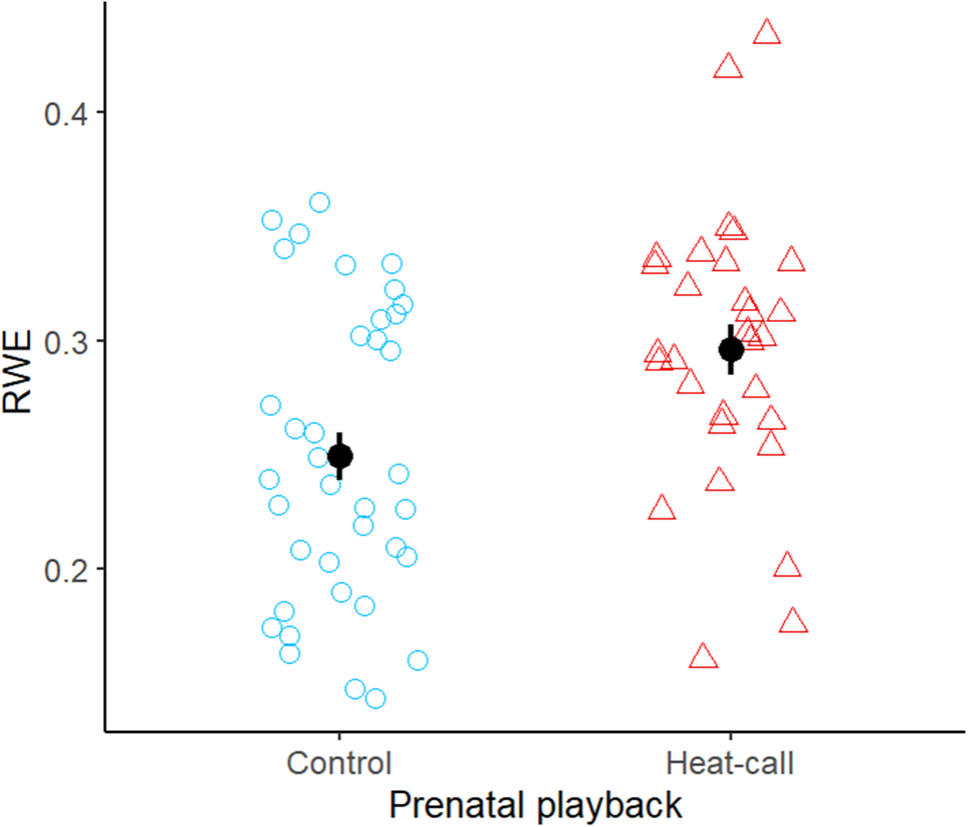
Effect of prenatal playback (control call vs heat-call) on relative water economy (RWE) at T_a_ = 27 °C (n = 67 for 34 birds), for birds prenatally exposed to either contact calls (control, blue dots) or heat-calls (red triangles). Markers and error bars show mean ± SE.

At thermoneutrality (i.e. T_a_ = 35 °C), treatment and control birds did not differ in any thermoregulatory variables (Table 1 and Supplementary Tables S7 and S8). However, below thermoneutrality (i.e. T_a_ = 27 °C), prenatal playback affected water balance as predicted: consistent with lower water requirements, RWE was significantly higher (and EHL/MHP significantly lower; Supplementary Table S8) in treatment birds (Table 1; Fig. 5), while EWL tended to be lower (p = 0.064, Table 1).

Lastly, in agreement with higher mass loss over the trial in the morning (see above), EWL was higher in the morning than afternoon (significantly at thermoneutrality, and marginally at T_a_ = 27 °C: p = 0.053), and morning RWE was significantly lower (at thermoneutrality: Table 1). At thermoneutrality, males also had higher EWL, EHL/MHP and lower RWE than females (Table 1 and Supplementary Table S8).

## Discussion

This study provides the first experimental evidence that prenatal acoustic experience affects avian thermoregulation at adulthood. In line with our hypothesis that heat-call exposure confers heat tolerance benefits at adulthood, heat-call individuals were more likely to reach T_a_ = 44 °C, at the time of day when respiratory evaporative cooling is most used (i.e. morning)^34,40^. This was, however, not achieved through reduced thermoregulation costs. Instead, MR and water loss at high T_a_ were higher in treatment than control birds, partly due to a stronger effect of activity on MR and evaporative cooling capacity. At low T_a_, nonetheless, prenatal heat-call exposure did shift individual adult phenotype towards greater water conservation, as predicted. Whereas mass loss (mainly reflecting cumulative water loss throughout the trial) was not significantly different, treatment birds replenished more of their body water post-trial, with most treatment individuals over-compensating for the mass loss in the morning, but not in the afternoon. Overall, our data demonstrate that prenatal exposure to parental heat-calls has multiple long-term effects on individual phenotypes. While more work is needed to establish the fitness impact under natural conditions, our findings suggest the adaptive benefits of heat-call exposure do not involve minimizing the costs of thermoregulation at high environmental temperature extremes, but instead improve water balance and heat tolerance.

Contrary to the notion of phenotypic plasticity lowering thermoregulation costs in summer-acclimatised and heat-acclimated birds^19,38,39^, we found that MR increased more steeply with air temperature in heat-call birds, to reach higher levels at T_a_ = 44 °C. This occurred as both activity and the associated increase in metabolic heat production were higher in treatment than control birds (even though activity levels during thermoregulatory measurements did not differ). Nevertheless, treatment birds performed better at high T_a_, as they were more likely to reach T_a_ = 44 °C in the morning (regardless of activity levels). Heat-call birds were therefore able to sustain higher costs of activity, without compromising heat tolerance, which may be beneficial to maintain foraging and breeding activities in hot weather. These effects on adult heat tolerance are also consistent with the previously demonstrated shift in thermal preferences towards hotter microsites at adulthood, and confirm that heat-calling to embryos may represent a novel mechanism for transgenerational heat adaptation^31^. More studies are nonetheless needed on the physiological impacts of activity under extreme heat. That this impact may be subject to developmental programming is particularly relevant in species such as the zebra finch, which, to avoid lethal dehydration^8,10^, must fly under extreme heat to drinking water (often several km away).

As predicted, heat-call exposed individuals conserved more water at mild T_a_ (T_a_ = 27 °C), as indicated by a higher water economy and lower EHL/MHP. No comparable differences were observed at or above thermoneutrality, possibly because individuals progressively started panting^16^, thus increasing the ratio of respiratory to cutaneous water loss^17^. Interestingly, cutaneous water loss has indeed been found to be sensitive to developmental conditions in other avian species^30,55^. For instance, acclimation to low humidity in house sparrow nestlings (*Passer domesticus indicus*) reduced fledglings’ cutaneous water loss by lowering the proportion of free fatty acid in the skin^55^. Whether prenatal acoustic signals increased water economy through a similar mechanism remains to be explored. A recent review nonetheless showed that developmental programming by prenatal sounds and vibrations is far more widespread across taxa than previously thought, including for physiological traits^31,36^. Here, we add to this evidence by demonstrating that prenatal acoustic communication affects long-term heat tolerance and water balance. That such inter-individual variation persisted well-into adulthood is important for understanding the strength of selection acting on these traits. Yet, to date, studies on developmental programming for heat tolerance had been restricted to the first few months of life, even in well-studied poultry (reviewed by Nord and Giroud^26^). To our knowledge, our study thus provides the first evidence for the long-term effects (> 6 months) of prenatal experience on endotherm heat tolerance or water balance.

Thermoregulation varied throughout the day, with EWL, EHL/MHP and mass loss higher, and RWE lower, in the morning than afternoon. This was expected, based on birds’ higher reliance on behaviours to enhance evaporative heat dissipation (e.g. panting and vocal panting), compared to behaviours maximising non-evaporative losses (e.g. wing spreading), earlier in the day, at a given T_a_^34,40,41,50^. Following our prediction, morning was also when differences between treatment and control birds were most pronounced, both in terms of the likelihood of reaching T_a_ = 44 °C and for mass recovery post-trial. Considering that foraging is often most intense in the morning^56^, including during reproduction^57^, improved heat tolerance at this time of day may particularly benefit fitness. This is particularly relevant considering the predicted impact of climate change on reproduction^9,58^.

Lastly, our data also add to the increasing literature on intra- and interspecific variation in avian thermoregulatory performance in the heat. Even though birds in our experiment remained well below the lethal dehydration threshold for small desert passerines (estimated at ~ 15% of body mass loss^8^), our values for RWE (< 1, indicating higher water loss than water gain from metabolic water production), support the view that zebra finches need to drink to maintain positive water balance^49,59^. Patterns of thermal physiology documented in the present study are similar to those reported at high temperatures in zebra finches^17,49,54^ and other small desert passerines in Australia^53,60^. These data, together with our finding of a heat tolerance limit close to T_a_ = 44 °C (even though thermal endpoints were not specifically elicited in our study), add to the evidence that, under the same experimental conditions, Australian arid-zone passerines generally possess lower heat tolerance than their counterparts from North America and southern Africa^10,53,61^ and non-passerine taxa that employ gular flutter or high rate of cutaneous evaporative heat dissipation^11^. Lastly, sex differences, with a higher T_b_ in females at high T_a_ and lower water loss at thermoneutrality, are particularly interesting. Indeed, the zebra finch lacks strong sexual dimorphism, and individuals were not breeding during the study (and therefore did not differ in immediate parental care activity). Overall, a better understanding of inter- and intraspecific sources of variation in thermal physiology is urgently needed to predict the global impact of climate change^10^.

In conclusion, we have demonstrated that exposure to a prenatal acoustic signal indicating hot conditions to embryos^31,34^ has long-term effects on the thermal phenotype of adult zebra finches. We found that prenatal heat-call exposure shifted adult phenotype toward higher water conservation in mild conditions, and improved their ability to sustain hot conditions at the most critical time of day (i.e. morning). Such acoustic experience also altered individuals’ activity and its metabolic impact, rather than minimizing thermoregulatory costs at high T_a_. These findings highlight the relevance of the acoustic channel to program offspring for long-term environmental conditions^31,33,36^, and provide a first line of evidence on the role of developmental programming in generating heat-adapted phenotypes in endotherms. Such inter-individual variation is paramount for rapid adaptation to climate change, particularly in desert environments where animals are already facing extreme conditions^10^.

## Materials and methods

All procedures were approved by Deakin University Animal Ethics Committee (G06-2017), the Animal Ethics Committee of the University of Pretoria (protocol EC048-18) and the Research and Scientific Ethics Committee of the South African National Biodiversity Institute (P18/36). All experiments were performed in accordance with Australian guidelines and regulations for the use of animals in research. This study was conducted in compliance with the ARRIVE guidelines (https://arriveguidelines.org).

### Experimental acoustic treatments and housing

Experimental birds were wild-derived zebra finches from an acoustic playback experiment previously presented in Mariette and Buchanan^31^. At laying (Feb–March 2014), eggs were collected from outdoor aviaries (Deakin University, Geelong, Australia), replaced by dummy eggs and placed in an artificial incubator at 37.5 °C and 60% relative humidity. After nine days, whole clutches were randomly assigned to one of two acoustic playback groups: treatment eggs were exposed to heat-calls (aka “incubation calls”) and controls to adult contact calls (i.e. *tet* calls), whilst both groups were also exposed to common nest-specific calls (i.e. whine calls) to ensure normal acoustic stimulation. Playbacks had 20 min of heat-calls or tet calls per 1h15, separated by silence and whine calls, and played from 9:30 a.m. to 6:30 p.m.^31^. To avoid any differences in incubation conditions, eggs and sound cards were swapped daily between incubators. After hatching, nestlings were reared in mixed or single-group broods, in the same outdoor aviaries (see Supplementary Material).

At adulthood (March–April 2018), we tested 34 experimental birds (16 females and 18 males; 15 treatment and 19 control birds) at the end of their fourth summer. From February 2018, birds were moved to indoor cages for acclimation, at least 27 days before experimental trials, at a constant room temperature of 25 °C and day-night cycle of 12 h:12 h, and supplied with ad libitum finch seed mix, grit, cucumber and water. After three days, we implanted a temperature-sensitive passive integrated transponder (PIT) tag (Biomark, Boise ID, USA) subcutaneously into the bird’s flank. Subcutaneous PIT tags reduce the risk of injuries and generally yield T_b_ values similar to those obtained using intraperitoneally-injected tags in small birds such as the zebra finch^62,63^.

### Experimental heat exposure protocol

All birds were tested twice. Each individual’s second trial occurred on a different day than the first, with an average of 16 days between the two trials, but each bird was tested in the morning for one trial (~ 10:30 a.m.) and in the afternoon (~ 2:50 p.m.) for the other, in random order. On average, trials lasted 125 min (range: 93–151 min). The predicted mean digesta retention time for a 12 g bird is ~ 50 min^64^. Hence, to ensure birds were post-absorptive, they were fasted (but with *ad-libitum* water) for two hours before each trial, within auditory and visual contact of conspecifics. Birds were then weighed to measure the initial mass (mass_init_ ± 0.01 g), before being placed individually in the metabolic chamber within a temperature-controlled cabinet. There were no significant difference in mass_init_ between heat-call (12.04 ± 0.18 g) and control individuals (12.03 ± 0.15 g; t (60) = − 0.059, p = 0.953).

During each trial, T_a_ in the metabolic chamber was gradually increased in a succession of “stages”. Trials started with T_a_ = 27 °C for 25 min or 45 min (for the first or second trial respectively), then T_a_ = 35 °C for 30 min (i.e. thermoneutrality^54^, followed by 20-min stages in succession at T_a_ = 40, 42 and 44 °C. Temperature transition took 1 (for 2 °C) to 6 min (for 8 °C increments).

To “complete the trial”, individuals had to be able to remain in the chamber for 20 min at T_a_ = 44 °C. Bird behaviour in the chamber was monitored using two infrared video cameras by an experimenter (AP) blind to playback treatments. The trial was terminated early if the bird showed sustained escape behaviour, or reached a thermal endpoint (e.g., loss of balance or severe hyperthermia with T_b_ > 45 °C^16,52^). Immediately after trial termination or completion, birds were taken out of the chamber and exposed to room temperature. They were then weighed (mass_end_), given water on their bill, and transferred to the holding room at 25 °C in an individual cage with ad libitum seeds and water. After one hour, birds were weighed again (mass_1h_). No bird died during the trials.

### Thermoregulatory measurements and data processing

We used an open flow-through respirometry system to measure CO_2_ production and EWL, following Whitfield et al.^52^ and as commonly used to assess avian thermoregulation in the heat^19,53,60^. Dry air was pushed into a 1.5-L plastic metabolic chamber, maintained at low humidity levels (< 0.72 kPa in excurrent air) by regulating the flow rate (range: 1–3.5 L.min^-1^) with a mass flow controller. Air was subsampled and pulled into H_2_O (RH-300, Sable Systems) and CO_2_ analysers (CA-10, Sable Systems). Details of the respirometry system and calibration procedures are in the Supplementary Material.

Following Whitfield et al.^52^, in Expedata, for each T_a_ stage, we selected the 1-min window with lowest and least variable CO_2_ and H_2_O values, after ≥ 10 min (or ≥ 5 min at T_a_ = 42–44 °C) of stable T_a_. We calculated MR and EWL using equations 9.5 and 9.6 from Lighton^65^, assuming a respiratory exchange ratio (RER) of 0.71 for fasted individuals^66^. Using a RER of 0.83 (i.e. metabolism of approximately equal mix of lipids and carbohydrates^60^ did not change any result. We computed relative water economy (RWE) as the ratio of metabolic water production (MWP; calculated from rates of CO_2_ production) to EWL^2,59^; and the evaporative cooling capacity as the ratio of EHL (calculated from EWL) to MHP (approximated by MR, see Supplementary Material)^67^. Body temperature was recorded every 10 s using a PIT tag reader, and averaged T_b_ calculated for the 1-min sampling window, accounting for 99% equilibrium time^68^ (6.9 min and 2 min for flow rates of 1 L min^−1^ and 3.5 L min^−1^, respectively).

Bird behaviour was monitored every 30 s and activity scored as: 0 = resting or sleeping, 1 = looking around while sitting mostly still, 2 = moving with no or small displacement by stepping, 3 = displacement usually by hopping, 4 = hopping repeatedly or jumping, 5 = sustained escape behaviour, jumping continuously. At each T_a_ stage, we averaged the activity (i) over the first 3 min at stable air temperature (activity_stage_) to test for inter-individual differences in activity levels under standard conditions, and (ii) over the 10 min prior and during measurement windows (activity_meas_) to account for current and carry-over effects of activity on metabolism (after equilibrium time^68^). Importantly, as per^52^, only data from calm birds were retained in analyses of thermoregulatory variables (i.e. here, activity ≤ 3 during the 1-min measurement, as well as the preceding 10 min).

We calculated mass loss over the trial (i.e. mass_init_-mass_end_) as a proxy for total water loss (including through defecation, as faeces contain 80% water^54^) and mass recovery post-trial as the percentage of mass loss regained after 1 h (i.e. [(mass_1h_ − mass_end_)/(mass_init_ − mass_end_)] * 100).

### Data analyses

All analyses were performed using R (v3.6.1) in RStudio (v1.1.1335). The total data set corresponded to 67 trials (n = 34 birds). One trial (out of 68) could not be used because the flow rate was set incorrectly. As data were restricted to calm birds, and some trials had to be terminated before reaching T_a_ = 44 °C, analyses were conducted on data from all 67 trials at T_a_ = 27, 35 and 40 °C, but 55 trials at T_a_ = 42 °C and 28 trials at T_a_ = 44 °C (Fig. 3). As on rare occasions the PIT tag angle or position prevented its detection by the antenna, sample sizes for T_b_ are n = 66 at T_a_ = 27 °C and n = 65 at T_a_ = 35 °C. For every model, predictors were centered and scaled and residuals checked for normality and homoscedasticity.

In all models (apart from segmented analyses), we tested for effects of prenatal playback, mass_init_, time-of-day (AM or PM), trial number (1st or 2nd trial) and sex as fixed factors, together with the interaction between playback and time-of-day, and with individual ID as a random factor. Non-significant interactions (p < 0.05) were not retained (full models are presented in Supplementary Material Tables S1–S8).

### Heat tolerance and body mass variation

The effect of prenatal playback on heat tolerance was assessed using two proxies as response variables: the maximum T_a_ reached (Max T_a_ = 42 or 44 °C, n = 67), and whether or not individuals reaching T_a_ = 44 °C (n = 53) completed the trial (i.e., spent 20 min at T_a_ = 44 °C). We fitted generalized linear mixed-effects models (GLMMs, *glmer* function from *lme4* R package) with a binomial error distribution and the fixed and random effects described above.

The effects on individual total water loss during the trial and subsequent body water replenishment in the following hour were investigated using two LMMs with predictors as described above and either mass loss (n = 67), or post-trial mass recovery (n = 66, as one individual was not weighed after 1 h), as response variables.

### Variation in activity throughout the trial

First, considering all birds, we tested how activity varied as a function of increasing T_a_. We defined the inflection point for activity_stage,_ for T_a_ ≥ 35 °C, using a Davies test and the function *segmented* from the *segmented* R package^69^. We then fitted linear mixed models (LMMs, *lmer* function from the *lme4* R package) above the inflection point, with prenatal playback, mass_init_, trial number, time-of-day, sex and the interaction between prenatal playback and recorded T_a_ as fixed effects, and trial nested within individual ID as random effects. Given the interaction was significant (see “Results”), we computed separate regression lines for treatment and control birds. Second, we tested for differences between prenatal playback groups on activity, separately at each T_a_ stage where thermoregulatory values were investigated: at the max T_a_ reached, T_a_ = 35 °C and T_a_ = 27 °C. We used LMMs, with activity_stage_ (i.e. activity in first 3 min at stable T_a_) or activity_meas_ (i.e. activity in the 10 min before and during metabolic measurements; square root transformed) as a response variable. Analyses on activity_stage_ were performed on all birds (except n = 12 when the stage lasted < 3 min at stable T_a_ before trial interruption), to test for overall playback effects (i.e. n = 55 at max T_a_ reached, n = 67 otherwise). Analyses on activity_meas_ however were restricted to calm birds only (i.e. activity scores ≤ 3), to match thermoregulatory analyses (i.e. n = 32 at max T_a_ and n = 67 otherwise). We used the same fixed and random factors as described above for all statistical analyses, in addition to T_a_ (= 42 or 44 °C) for analyses at the max T_a_ reached only.

To establish if there were any bias in trial termination criteria between playback groups (even though the observer was blind to treatment), we tested for differences in activity level during the last 3 min at T_a_ = 42 °C for birds reaching T_a_ = 44 °C, (i.e. activity_42-end_, n = 53 trials). We fitted a LMM with predictors and random effect as described above. Activity_42-end_ was square root transformed to meet linear model assumptions.

### Thermoregulatory responses above thermoneutral zone

To investigate individual overall thermoregulatory response to heat, we first defined the upper limit of thermoneutrality (i.e. increase in MR) and inflection points for other variables (EWL, T_b,_ RWE, EHL/MHP) for T_a_ ≥ 35 °C, using a Davies test and *segmented* function, as described above for activity_stage_. This was then again followed by LMMs above the respective inflection points, with predictors, random effect and interaction as above.

To examine responses at the most extreme T_a_ stage reached (i.e. Max T_a_ = 42 or 44 °C, n = 32 trials with measurements on calm individuals), we fitted LMMs on MR, EWL, T_b_, RWE and EHL/MHP, with predictors as described above. We included Max T_a_ (42 or 44 °C) as an additional fixed factor, and activity_meas_ as a covariate and in interaction with playback, to account for potential activity effects on thermoregulatory values.

### Thermoregulatory response at mild temperatures

We examined the effect of playback on each thermoregulatory value (MR, EWL, T_b,_ RWE and EHL/MHP) (i) at thermoneutrality (T_a_ = 35 °C, n = 67 trials) and (ii) at mild T_a_ (T_a_ = 27 °C, n = 67 trials) using LMMs, with predictors as described above and activity_meas_ and its interaction with playback.

## Supplementary Information

Supplementary Information.

## Data Availability

Datasets used in this manuscript are available from the Mendeley Data Repository: https://data.mendeley.com/datasets/t45rjhtk9w/draft?a=9fa8157f-69fa-4753-a5b2-6ed24a5a28be.

## References

[1] McKechnie AE, Dunn PO, Moller AP (2019). Physiological and morphological effects of climate change. Effects of Climate Change on Birds.

[2] Withers PC, Cooper CE, Maloney SK, Bozinovic F, Cruz-Neto AP (2016). Ecological and Environmental Physiology of Mammals.

[3] Du Plessis KL, Martin RO, Hockey PA, Cunningham SJ, Ridley AR (2012). The costs of keeping cool in a warming world: Implications of high temperatures for foraging, thermoregulation and body condition of an arid-zone bird. Glob. Chang. Biol..

[4] Tapper S, Nocera JJ, Burness G (2020). Heat dissipation capacity influences reproductive performance in an aerial insectivore. J. Exp. Biol..

[5] Nilsson J-Å, Nord A (2018). Testing the heat dissipation limit theory in a breeding passerine. Proc. R. Soc..

[6] Wolf B (2000). Global warming and avian occupancy of hot deserts; a physiological and behavioral perspective. Rev. Chil. Hist. Nat..

[7] IPCC. *Climate change 2014: synthesis report. *In *Contribution of Working Groups I, II and III to the fifth assessment report of the Intergovernmental Panel on Climate Change* (eds Core Writing Team *et al.*) 151 (IPCC, 2014).

[8] Albright TP (2017). Mapping evaporative water loss in desert passerines reveals an expanding threat of lethal dehydration. PNAS.

[9] Conradie SR, Woodborne SM, Cunningham SJ, McKechnie AE (2019). Chronic, sublethal effects of high temperatures will cause severe declines in southern African arid-zone birds during the 21st century. PNAS.

[10] Conradie SR (2020). Avian mortality risk during heat waves will increase greatly in arid Australia during the 21st century. Conserv. Physiol..

[11] McKechnie AE, Wolf BO (2019). The physiology of heat tolerance in small endotherms. Physiology.

[12] Fuller A, Hetem RS, Maloney SK, Mitchell D (2014). Adaptation to heat and water shortage in large, arid-zone mammals. Physiology.

[13] Gerson AR (2019). The functional significance of facultative hyperthermia varies with body size and phylogeny in birds. Funct. Ecol..

[14] Glanville EJ, Murray SA, Seebacher F (2012). Thermal adaptation in endotherms: Climate and phylogeny interact to determine population-level responses in a wild rat. Funct. Ecol..

[15] Smit B, McKechnie AE (2010). Avian seasonal metabolic variation in a subtropical desert: Basal metabolic rates are lower in winter than in summer. Funct. Ecol..

[16] Pessato A, McKechnie AE, Buchanan KL, Mariette MM (2020). Vocal panting: A novel thermoregulatory mechanism for enhancing heat tolerance in a desert-adapted bird. Sci. Rep..

[17] Wojciechowski MS, Kowalczewska A, Colominas-Ciuró R, Jefimow M (2020). Phenotypic flexibility in heat production and heat loss in response to thermal and hydric acclimation in the zebra finch, a small arid-zone passerine. J. Comp. Physiol. B.

[18] Williams JB, Tieleman BI (2000). Flexibility in basal metabolic rate and evaporative water loss among hoopoe larks exposed to different environmental temperatures. J. Exp. Biol..

[19] Noakes MJ, McKechnie AE (2019). Reaction norms for heat tolerance and evaporative cooling capacity do not vary across a climatic gradient in a passerine bird. Comp. Biochem. Physiol. A.

[20] Catullo RA, Llewelyn J, Phillips BL, Moritz CC (2019). The potential for rapid evolution under anthropogenic climate change. Curr. Biol..

[21] Noble DW, Radersma R, Uller T (2019). Plastic responses to novel environments are biased towards phenotype dimensions with high additive genetic variation. PNAS.

[22] Monaghan P (2008). Early growth conditions, phenotypic development and environmental change. Philos. Trans. R. Soc. B.

[23] Mousseau TA, Fox CW (1998). The adaptive significance of maternal effects. TREE.

[24] Bull J, Vogt RC (1979). Temperature-dependent sex determination in turtles. Science.

[25] Donelson J, Munday P, McCormick M, Pitcher C (2012). Rapid transgenerational acclimation of a tropical reef fish to climate change. Nat. Clim. Change.

[26] Nord A, Giroud S (2020). Lifelong effects of thermal challenges during development in birds and mammals. Front. Physiol..

[27] Piestun Y, Druyan S, Brake J, Yahav S (2013). Thermal manipulations during broiler incubation alter performance of broilers to 70 days of age. Poult. Sci..

[28] Saleh KM, Tarkhan AH, Al-Zghoul MB (2020). Embryonic thermal manipulation affects the antioxidant response to post-hatch thermal exposure in broiler chickens. Animals.

[29] Andreasson F, Nord A, Nilsson J-Å (2018). Experimentally increased nest temperature affects body temperature, growth and apparent survival in blue tit nestlings. J. Avian. Biol..

[30] Marder J, Arieli Y (1988). Heat balance of acclimated pigeons (*Columba livia*) exposed to temperatures up to 60 °C Ta. Comp. Biochem. Physiol. A.

[31] Mariette MM, Buchanan KL (2016). Prenatal acoustic communication programs offspring for high posthatching temperatures in a songbird. Science.

[32] Mariette MM (2019). Acoustic cooperation: Acoustic communication regulates conflict and cooperation within the family. Front. Ecol. Evol..

[33] Mariette MM, Clayton DF, Buchanan KL (2021). Acoustic developmental programming: A mechanistic and evolutionary framework. TREE.

[34] Mariette MM (2018). Parent-embryo acoustic communication: A specialised heat vocalisation allowing embryonic eavesdropping. Sci. Rep..

[35] Mariette MM, Buchanan KL (2019). Calling in the heat: The zebra finch incubation call depends on heat AND reproductive stage—A comment on McDiarmid et al. 2018. Behav. Ecol..

[36] Noguera JC, Velando A (2019). Bird embryos perceive vibratory cues of predation risk from clutch mates. Nat. Ecol. Evol..

[37] Noguera JC, Velando A (2019). Reduced telomere length in embryos exposed to predator cues. J. Exp. Biol..

[38] Noakes MJ, Wolf BO, McKechnie AE (2016). Seasonal and geographical variation in heat tolerance and evaporative cooling capacity in a passerine bird. J. Exp. Biol..

[39] Williams JB, Tieleman BI (2005). Physiological adaptation in desert birds. Bioscience.

[40] Grant, G. S. Avian incubation: Egg temperature, nest humidity, and behavioral thermoregulation in a hot environment. *Ornithol. Monogr.* iii-75 (1982).

[41] Xie S, Turrell EJ, McWhorter TJ (2017). Behavioural responses to heat in captive native Australian birds. Emu.

[42] Tieleman BI, Williams JB, Buschur ME (2002). Physiological adjustments to arid and mesic environments in larks (Alaudidae). Physiol. Biochem. Zool..

[43] Scott GR, Johnston IA (2012). Temperature during embryonic development has persistent effects on thermal acclimation capacity in zebrafish. PNAS.

[44] Kellermann V, Sgrò CM (2018). Evidence for lower plasticity in CTMAX at warmer developmental temperatures. J. Evol. Biol..

[45] Gunderson AR, Fargevieille A, Warner DA (2020). Egg incubation temperature does not influence adult heat tolerance in the lizard Anolis sagrei. Biol. Lett..

[46] Abayarathna T, Murray BR, Webb JK (2019). Higher incubation temperatures produce long-lasting upward shifts in cold tolerance, but not heat tolerance, of hatchling geckos. BiO.

[47] Ben-Ezra N, Burness G (2017). Constant and cycling incubation temperatures have long-term effects on the morphology and metabolic rate of Japanese Quail. Physiol. Biochem. Zool..

[48] Burness G, Huard JR, Malcolm E, Tattersall GJ (2013). Post-hatch heat warms adult beaks: Irreversible physiological plasticity in Japanese quail. Proc. R. Soc..

[49] Cade TJ, Tobin CA, Gold A (1965). Water economy and metabolism of two estrildine finches. Physiol. Zool..

[50] Smit B, Harding C, Hockey PA, McKechnie AE (2013). Adaptive thermoregulation during summer in two populations of an arid-zone passerine. Ecology.

[51] Careau V, Thomas D, Humphries M, Réale D (2008). Energy metabolism and animal personality. Oikos.

[52] Whitfield MC, Smit B, McKechnie AE, Wolf BO (2015). Avian thermoregulation in the heat: Scaling of heat tolerance and evaporative cooling capacity in three southern African arid-zone passerines. J. Exp. Biol..

[53] McKechnie AE (2017). Avian thermoregulation in the heat: Evaporative cooling in five Australian passerines reveals within-order biogeographic variation in heat tolerance. J. Exp. Biol..

[54] Calder WA (1964). Gaseous metabolism and water relations of the zebra finch, *Taeniopygia castanotis*. Physiol. Zool..

[55] Muñoz-Garcia A, Williams JB (2008). Developmental plasticity of cutaneous water loss and lipid composition in stratum corneum of desert and mesic nestling house sparrows. PNAS.

[56] Cooper CE, Withers P, Hurley L, Griffith SC (2019). The field metabolic rate, water turnover and feeding and drinking behaviour of a small avian desert granivore. Front. Physiol..

[57] Mariette MM (2011). Using an electronic monitoring system to link offspring provisioning and foraging behavior of a wild passerine. Auk.

[58] Bourne AR, Cunningham SJ, Spottiswoode CN, Ridley AR (2020). High temperatures drive offspring mortality in a cooperatively breeding bird. Proc. R. Soc..

[59] Cooper CE, Hurley LL, Deviche P, Griffith SC (2020). Physiological responses of wild zebra finches (*Taeniopygia guttata*) to heatwaves. J. Exp. Biol..

[60] Smith EK, O'Neill JJ, Gerson AR, McKechnie AE, Wolf BO (2017). Avian thermoregulation in the heat: Resting metabolism, evaporative cooling, and heat tolerance in Sonoran Desert songbirds. J. Exp. Biol..

[61] Conradie SR (2022). Global heating poses a serious threat to Australia’s birds: Reply to Pacheco-Fuentes et al.. Conserv. Physiol..

[62] Oswald KN, Evlambiou AA, Ribeiro ÂM, Smit B (2018). Tag location and risk assessment for passive integrated transponder-tagging passerines. Ibis.

[63] McCafferty DJ, Gallon S, Nord A (2015). Challenges of measuring body temperatures of free-ranging birds and mammals. Anim. Biotelemetry.

[64] Karasov WH (1990). Digestion in birds: Chemical and physiological determinants and ecological implications. Stud. Avian Biol..

[65] Lighton JR (2008). Measuring Metabolic Rates: A Manual for Scientists.

[66] Walsberg G, Wolf B (1995). Variation in the respiratory quotient of birds and implications for indirect calorimetry using measurements of carbon dioxide production. J. Exp. Biol..

[67] Tracy RL, Walsberg GE (2001). Developmental and acclimatory contributions to water loss in a desert rodent: Investigating the time course of adaptive change. J. Comp. Physiol. B.

[68] Lasiewski RC, Acosta AL, Bern-Stein MH (1966). Evaporative water loss in birds. 1. Characteristics of the open flow method of determination, and their relation to estimates of thermoregulatory ability. Comp. Biochem. Physiol. A.

[69] Muggeo VM (2008). Segmented: An R package to fit regression models with broken-line relationships. R News.

