# Supplementary material for: A prenatal acoustic signal of heat affects thermoregulation capacities at adulthood in an arid-adapted bird
Source: Sci Rep. 2022 Apr 7;12:5842. doi: 10.1038/s41598-022-09761-1 (PMC8991222; doi:10.1038/s41598-022-09761-1)
Supplement: Supplementary file 1 — Supplementary Information. [file 41598_2022_9761_MOESM1_ESM.pdf]

A prenatal acoustic signal of heat affects thermoregulation capacities at adulthood in an arid-adapted bird.

Anaïs Pessato[1*]

Andrew E. McKechnie[2, 3]

Mylene M. Mariette[1, 4*]

[1]Centre for Integrative Ecology, School of Life & Environmental Sciences, Deakin University, Geelong 3216, Australia.

[2]South African Research Chair in Conservation Physiology, South African National Biodiversity Institute, Pretoria 0001, South Africa

[3]DSI-NRF Centre of Excellence at the FitzPatrick Institute, Department of Zoology and Entomology, University of Pretoria, Pretoria 0001, South Africa

[4]Doñana Biological Station EBD-CSIC, 41092 Seville, Spain

*Corresponding authors

apessato@deakin.edu.au
m.mariette@deakin.edu.au

Supplementary information

Supplementary materials and methods

Experience from hatching to test at adulthood

After hatching, nestlings were returned to either their parents or foster parents in mixed broods (n=18 birds) or single prenatal playback broods (n=16 birds) in shared outdoor aviaries. Mixed broods allow controlling for shared postnatal environment but cannot occur naturally; incubating parents cannot signal to only half of the clutch and therefore would not normally rear mixed broods). Before sexual maturity, offspring (both control and treatment groups) were moved together to another aviary where they all experienced natural weather conditions (from 2014 to 2018: mean min temperature= 4.7°C, mean max temperature=27.6°C, weather data obtained from Bureau of Meteorology, www.bom.gov.au). There was no difference in survival between treatment and control groups at early stages[1].

At adulthood, birds were tested during daytime, when they would normally experience high air temperatures. To lower the possible stress due to social isolation in the metabolic chamber during the trial, familiar acoustic background from birds in the holding room was broadcast (as routinely done in behavioural experiments[2] with a Bluetooth speaker (QUDO Splashproof Bluetooth Speaker, officeworks LTD, Australia).

Open flow-through respirometry system and calibration of equipment

We used a pump (Gast DAA-V515-ED, Cole-Parmer) to push dry air (scrubbed with silica gel and Drierite ®) into the metabolic chamber (1.5 L, plastic clip container, 8 x 18 x 11.5 cm). The air in the metabolic chamber was maintained with at low humidity level by regulating the airflow with a mass flow controller (Alicat scientific Inc., USA). Mass flow controller was calibrated and accurate within 0.008 SPLM within the range we used (1 to 3.5 $L.min^{-1}$). Baseline air was subsampled between each T_a stage for five minutes. Baseline and excurrent

chamber air were subsampled and pulled by a pump (SS4 subsampler, Sable Systems) at 213 $mL.min^{-1}$ through the H_2O analyser (RH-300, Sable Systems) and CO_2 analyser (CA-10, Sable Systems). H_2O analyser was calibrated daily using the O_2 dilution technique[3] and the CO_2 analyser every second day using certified gas with a known CO_2 concentration of 2080 ppm (BOC, Australia). Both of the analysers were zeroed with pure nitrogen (Nitrogen 5.0; 99.999%, Supagas, Australia). Gas concentrations and temperatures were recorded via an analog-digital converter (UI2, Sable Systems) and Expedata software (Sable Systems).

During the trial, body temperature was recorded every 10 sec using a PIT tag reader (HPR plus, Biomark, Boise ID, 172 USA). No aberrant values were recorded; all values during the one-minute measure were thus averaged. A subset of PIT tags were calibrated in a water bath against a type T thermocouple (BAT-12, Physitemp Instruments Inc., USA) at water temperature ranging from 40°C to 48°C. PIT tags were accurate within 0.27 ± 0.03°C.

Data processing and statistical analyses

Relative water economy (RWE) was computed as MWP/EWL with MWP calculated from rates of CO_2 production assuming 0.801 mg H_2O mL^{-1} CO_2 [4]. We also computed the evaporative cooling capacity (EHL/MHP) as EHL/MR. We converted rates of EWL to heat loss (EHL in W) assuming a latent heat of vaporisation of 2.4 J mg^{-1} H_2O [5].

In all analyses, we did not include clutch or brood ID because the variance for clutch or brood ID was often close to zero. This is likely because over half of the individuals in this experiment did not have siblings (20 individuals without genetic siblings out of 26 clutches, and 14 individuals without foster siblings out of 22 broods), due to normal mortality of embryos, hatchlings and juveniles (as well as adult mortality).

Supplementary tables

	Max T_a reached			Complete trial		
Predictors	*Est.*	*SE*	*p*	*Est.*	*SE*	*p*
Reduced model						
Intercept	17.544	8.997	0.051	-12.181	6.796	0.073
Playback	13.654	7.540	0.070	-3.057	3.755	0.416
$Mass_{init}$	-0.076	1.891	0.968	-1.715	1.820	0.346
Time	4.256	3.180	0.181	-4.595	3.046	0.132
Trial	-3.747	3.343	0.262	3.572	2.745	0.193
Sex	-1.833	3.866	0.636	0.337	3.760	0.929
Playback x time	-18.718	9.062	**0.039**			
Full model						
Intercept	17.544	8.997	0.051	-12.163	7.513	**0.105**
Playback	13.654	7.540	0.070	-3.052	3.795	0.421
$Mass_{init}$	-0.076	1.891	0.968	-1.714	1.861	0.357
Time	4.256	3.180	0.181	-4.587	3.567	0.198
Trial	-3.747	3.343	0.262	3.564	3.359	0.289
Sex	-1.833	3.866	0.636	0.334	3.769	0.929
Playback x time	-18.718	9.062	**0.039**	0.001	4.885	1

Table S1: Outputs of the mixed effect models on heat tolerance variables: maximum T_a reached (42 or 44°C, considering all birds, n=67) and the ability to complete trial (i.e. staid 20 minutes at T_a=44°C) among birds reaching T_a=44°C (n=53). The reference is the control group for playback, morning for time-of-day, here “Time”, trial 1 for trial and female for sex. Est. ± SE corresponds to estimate ± standard error. Bold font shows significant effect (p<0.05).

	**Mass loss**			**Mass recovery post-trial**		
Predictors	*Est.*	*SE*	*p*	*Est.*	*SE*	*p*
Reduced model						
Intercept	0.576	0.073	**<0.001**	55.301	20.472	**0.009**
Playback	-0.045	0.062	0.475	42.007	17.436	**0.020**
$Mass_{init}$	0.045	0.028	0.108	-16.049	7.162	**0.030**
Time	-0.061	0.029	**0.041**	5.050	12.373	0.686
Trial	-0.001	0.030	0.968	18.047	9.748	0.073
Sex	0.085	0.062	0.180	16.447	14.748	0.273
Playback x time				-64.761	18.659	**0.002**
Full Model						
Intercept	0.565	0.073	**<0.001**	55.301	20.472	**0.009**
Playback	-0.009	0.069	0.901	42.007	17.436	**0.020**
$Mass_{init}$	0.046	0.028	0.100	-16.049	7.162	**0.030**
Time	-0.029	0.038	0.452	5.050	12.373	0.686
Trial	-0.005	0.030	0.560	18.047	9.748	0.073
Sex	0.086	0.062	0.176	16.447	14.748	0.273
Playback x time	-0.072	0.056	0.208	-64.761	18.659	**0.002**

Table S2: Outputs of mixed effect models on mass loss (n=67 for 34 birds) and mass recovery post-trial (n=66 for 34 birds). The reference is the control group for playback, morning for time-of-day, here “Time”, trial 1 for trial and female for sex. Est. ± SE corresponds to estimate ± standard error. Bold font shows significant effect ($p<0.05$).

	Sqrt activity$_{stage}$ (3-minutes)							
	**Above inflection pt**		**Max T_a reached**		**T_a =35°C**		**T_a =27°C**	
Predictors	*Est.*±SE	*p*	*Est.*±SE	*p*	*Est.*.±SE	*p*	*Est.*.±SE	*p*
Reduced model								
Intercept	1.184±0.131	**<0.001**	1.733±0.233	**<0.001**	0.560±0.138	**<0.001**	0.301±0.135	**0.029**
Playback	0.002±0.104	0.985	0.153±0.144	0.296	-0.018±0.089	0.838	-0.045±0.086	0.600
Mass$_{init}$	0.031±0.049	0.535	-0.046±0.069	0.501	0.008±0.044	0.862	0.095±0.043	**0.032**
Time	0.019±0.056	0.740	0.002±0.094	0.984	-0.010±0.068	0.0889	0.150±0.067	**0.032**
Trial	-0.126±0.059	**0.039**	-0.212±0.095	**0.035**	-0.097±0.070	0.173	-0.040±0.068	0.565
Sex	0.014±0.104	0.895	-0.123±0.146	0.408	-0.080±0.089	0.378	0.120±0.086	0.173
T_a	0.279±0.037	**<0.001**	-0.033±0.155	0.834	/	/	/	/
Playb x Time	0.132±0.056	**0.021**						
Full model								
Intercept	1.184±0.131	**<0.001**	0.132±0.056	**0.021**	0.598±0.141	**<0.001**	0.265±0.132	**0.050**
Playback	0.002±0.104	0.985	0.0201±0.173	0.251	-0.044±0.113	0.699	0.083±0.107	0.444
Mass$_{init}$	0.031±0.049	0.535	-0.046±0.069	0.509	0.008±0.044	0.858	0.099±0.043	**0.026**
Time	0.019±0.056	0.740	0.045±0.129	0.729	-0.032±0.093	0.730	0.262±0.085	**0.004**
Trial	-0.126±0.059	**0.039**	-0.209±0.097	**0.040**	-0.095±0.071	0.193	-0.055±0.066	0.411
Sex	0.014±0.104	0.895	-0.123±0.147	0.411	-0.080±0.089	0.375	0.122±0.086	0.165
T_a	0.279±0.037	**<0.001**	-0.049±0.158	0.759	/	/	/	/
Playb x Time	0.132±0.056	**0.021**	-0.094±0.190	0.625	0.051±0.138	0.717	-0.253±0.127	0.056

Table S3: Outputs of mixed effect models on activity$_{stage}$ (during the 3 first minutes at a T_a stage) on all birds tested (unless the stage lasted <3min before trial termination): above inflection point (n=146 observations for 34 birds), at max T_a reached (n=55 for activity$_{stage}$), T_a=35°C and T_a=27°C (n=67). The reference is the control group for playback, morning for time-of-day, here "Time", trial 1 for trial and female for sex. Est. ± SE corresponds to estimate ± standard error. Bold font shows significant effect (p<0.05).

Sqrt activity$_{42\text{-end}}$						
	Reduced model			***Full model***		
Predictors	*Est.*	*SE*	*p*	*Est.*	*SE*	*p*
Intercept	1.039	0.247	**<0.001**	0.995	0.256	**<0.001**
Playback	0.184	0.160	0.260	0.261	0.191	0.179
Mass$_{init}$	0.048	0.079	0.547	0.046	0.079	0.565
Time	0.143	0.110	0.209	0.218	0.154	0.171
Trial	-0.0156	0.116	0.192	-0.153	0.119	0.210
Sex	0.021	0.159	0.897	0.018	0.157	0.911
Playb x Time				-0.164	0.222	0.468

Table S4: Outputs of mixed effect models on activity$_{42\text{-end}}$ (during the last 3 minutes at a T_a=42°C, on all birds reaching T_a=44°C, n=53). The reference is the control group for playback, morning for time-of-day, here "Time", trial 1 for trial and female for sex. Est. ± SE corresponds to estimate ± standard error. Bold font shows significant effect ($p<0.05$).

	Activity$_{meas}$					
	**Max T_a reached**		**T_a =35°C**		**T_a =27°C**	
Predictors	*Est.*±SE	*p*	*Est.*±SE	*p*	*Est.*.±SE	*p*
Reduced model						
Intercept	2.792±0.624	**<0.001**	0.208±0.057	**<0.001**	0.625±0.074	**<0.001**
Playback	0.201±0.257	0.447	-0.005±0.035	0.879	-0.055±0.049	0.273
Mass$_{init}$	-0.066±0.126	0.604	0.025±0.018	0.173	0.012±0.024	0.628
Time	0.095±0.233	0.689	-0.024±0.028	0.412	-0.006±0.036	0.864
Trial	-0.706±0.235	**0.009**	-0.015±0.029	0.616	-0.203±0.037	**<0.001**
Sex	-0.008±0.272	0.976	0.014±0.035	0.693	-0.010±0.049	0.842
T_a	-0.433±0.403	0.299	/	/	/	/
Full model						
Intercept	2.626±0.642	**<0.001**	0.213±0.058	**<0.001**	0.626±0.076	**<0.001**
Playback	0.390±0.314	0.226	-0.022±0.046	0.641	-0.055±0.061	0.372
Mass$_{init}$	-0.063±0.125	0.620	0.025±0.018	0.175	0.012±0.024	0.634
Time	0.309±0.310	0.341	-0.038±0.039	0.335	-0.007±0.049	0.892
Trial	-0.647±0.241	**0.018**	-0.013±0.030	0.664	-0.203±0.038	**<0.001**
Sex	-0.046±0.273	0.868	0.014±0.035	0.700	-0.010±0.049	0.841
T_a	-0.427±0.401	0.303	/	/	/	/
Playb x Time	-0.507±0.485	0.315	0.032±0.058	0.587	0.001±0.073	0.989

Table S5: Outputs of mixed effect models on activity$_{meas}$ (10 minutes before and during stable thermoregulatory measurements) at max T_a reached among the calm individuals from which we obtained thermoregulatory values (n=32 for activiy$_{meas}$), T_a=35°C and T_a=27°C (n=67). The reference is the control group for playback, morning for time-of-day, here "Time", trial 1 for trial and female for sex. Est. ± SE corresponds to estimate ± standard error. Bold font shows significant effect (p<0.05).

	MR		EWL		T_b		RWE	
Predictors	Est. ± SE	p	Est. ± SE	p			Est. ± SE	p
Reduced model above inflection points								
Intercept	0.271±0.027	**<0.001**	9.200±0.456	**<0.001**	43.697 ±0.209	**<0.001**	0.051±0.004	**<0.001**
Playback	0.024±0.014	0.088	0.124±0.269	0.648	0.030±0.167	0.860	0.002±0.002	0.286
$Mass_{init}$	0.007±0.007	0.346	0.257±0.137	0.071	-0.031±0.075	0.685	<0.001±0.001	0.986
Time	0.005±0.014	0.732	-0.230±0.217	0.318	0.171±0.079	**0.042**	0.003±0.002	0.097
Trial	0.003±0.014	0.852	-0.489±0.233	**0.042**	-0.113±0.090	0.218	0.004±0.002	**0.023**
Sex	0.008±0.014	0.561	0.175±0.272	0.525	-0.218±0.169	0.208	<0.001±0.002	0.919
T_a	0.012±0.008	0.138	1.120±0.134	**<0.001**	0.380±0.050	**<0.001**	-0.004±0.001	**<0.001**
$Activity_{meas}$	0.030±0.007	**<0.001**	0.283±0.125	**0.027**	0.336±0.056	**<0.001**	0.004±0.001	**<0.001**
Playb x T_a	0.040±0.012	**0.002**	0.396±0.191	**0.044**				
Full model above inflection points								
Intercept	0.271±0.027	**<0.001**	9.200±0.456	**<0.001**	43.690 ±0.207	**<0.001**	0.051±0.004	**<0.001**
Playback	0.024±0.014	0.088	0.124±0.269	0.648	0.038±0.166	0.820	0.003±0.002	0.236
$Mass_{init}$	0.007±0.007	0.346	0.257±0.137	0.071	-0.036±0.075	0.631	<-0.001±0.001	0.958
Time	0.005±0.014	0.732	-0.230±0.217	0.318	0.173±0.078	**0.037**	0.003±0.002	0.078
Trial	0.003±0.014	0.852	-0.489±0.233	**0.042**	-0.112±0.089	0.217	0.004±0.002	**0.021**
Sex	0.008±0.014	0.561	0.175±0.272	0.525	-0.221±0.168	0.200	0.001±0.002	0.790
T_a	0.012±0.008	0.138	1.120±0.134	**<0.001**	0.335±0.057	**<0.001**	-0.005±0.001	**<0.001**
$Activity_{meas}$	0.030±0.007	**<0.001**	0.283±0.125	**0.027**	0.330±0.055	**<0.001**	0.004±0.001	**<0.001**
Playb. x T_a	0.040±0.012	**0.002**	0.396±0.191	**0.044**	0.120±0.073	0.110	0.003±0.002	0.107

Table S6: Outputs of linear mixed models above T_a inflection points (i.e. corresponding mainly to the three T_a stages at 40°C and above) for metabolic rate (i.e. MR, n=83 observations for 32 birds), evaporative water loss (i.e. EWL, n=83 observations for 32 birds), body temperature (i.e. T_b, n=94 observations for 32 birds) and relative water economy (i.e. RWE, n=83 observations for 32 birds) of calm birds (i.e. $acitivy_{meas} \leq 3$). Bold font corresponds to significant effect (p<0.05). The reference is the control group for playback, morning for time-of-day, here "Time", trial 1 for trial and female for sex. Est. ± SE corresponds to estimate ± standard error. Bold font shows significant effect (p<0.05).

Predictors	*Est. ± SE*	*p*	*Est. ± SE*	*p*	*Est. ± SE*	*p*	*Est. ± SE*	*p*
	**MR**		**EWL**		**RWE**		**T_b**	
**Max T_a (42°C or 44°C)**								
Intercept	0.247±0.092	**0.013**	9.497±1.201	**<0.001**	0.049±0.009	**<0.001**	44.085±0.489	**<0.001**
Playback	0.048±0.042	0.268	0.281±0.551	0.615	0.004±0.004	0.307	0.063±0.229	0.785
Mass	0.018±0.016	0.282	0.225±0.218	0.316	0.003±0.002	0.097	-0.018±0.095	0.854
Time	0.006±0.044	0.900	-0.330±0.568	0.572	0.003±0.004	0.415	0.074±0.207	0.726
Trial	0.027±0.038	0.480	-1.011±0.490	0.056	0.010±0.003	**0.012**	-0.231±0.185	0.230
$Activity_{meas}$	0.023±0.022	0.313	0.251±0.292	0.399	0.003±0.002	0.182	0.391±0.119	**0.003**
Sex	0.017±0.034	0.631	0.300±0.461	0.527	0.001±0.004	0.749	-0.603±0.209	**0.013**
T_a	0.015±0.052	0.775	2.230±0.695	**0.006**	-0.013±0.005	**0.037**	0.502±0.309	0.125
Playb. x time	0.038±0.067	0.576	0.826±0.855	0.353	0.002±0.006	0.693	0.304±0.311	0.349
Playb.x act_{meas}	0.076±0.036	**0.046**	0.474±0.465	0.320	0.007±0.003	**0.044**	0.055±0.177	0.760
**T_a= 35°C**								
Intercept	0.218±0.018	**<0.001**	0.230±0.431	**<0.001**	0.181±0.019	**<0.001**	41.073±0.270	**<0.001**
Playback	0.005±0.017	0.751	0.153±0.409	0.709	-0.001±0.018	0.941	0.050±0.273	0.855
Mass	0.016±0.007	**0.020**	0.296±0.164	0.076	-0.003±0.007	0.722	0.152±0.098	0.127
Time	-0.005±0.009	0.554	-0.336±0.222	0.142	0.024±0.09	**0.016**	0.039±0.115	0.736
Trial	-0.002±0.007	0.789	-0.092±0.174	0.602	0.003±0.007	0.677	0.132±0.091	0.154
$Activity_{meas}$	-0.007±0.006	0.274	-0.181±0.152	0.240	0.002±0.006	0.750	-0.020±0.078	0.804
Sex	0.012±0.015	0.435	0.798±0.372	**0.041**	-0.034±0.016	**0.044**	0.012±0.258	0.962
Playb. x time	0.001±0.013	0.915	-0.204±0327	**0.539**	-0.004±0.014	0.785	-0.059±0.163	0.722
Playb.x act_{meas}	<-0.001±0.009	0.998	0.285±0.233	0.229	-0.010±0.010	0.299	0.053±0.121	0.664
**T_a = 27°C**								
Intercept	0.367±0.024	**<0.001**	3.099±0.324	**<0.001**	0.204±0.030	**<0.001**	41.879±0.369	**<0.001**
Playback	0.010±0.021	0.649	-0.318±0.276	0.256	0.034±0.021	0.113	0.246±0.326	0.454
Mass	0.020±0.008	**0.018**	0.133±0.110	0.230	-0.001±0.009	0.928	0.176±0.126	0.168
Time	-0.007±0.010	0.476	-0.061±0.141	0.670	0.006±0.016	0.718	0.127±0.155	0.421
Trial	-0.007±0.010	0.473	-0.425±0.147	**0.006**	0.039±0.015	**0.014**	-0.438±0.164	**0.011**
$Activity_{meas}$	0.010±0.007	0.171	0.073±0.098	0.460	-0.001±0.010	0.945	0.199±0.109	0.076
Sex	0.002±0.019	0.905	0.433±0.252	0.096	-0.031±0.017	0.090	-0.115±0.296	0.701
Playb. x time	-0.013±0.014	0.376	-0.362±0.210	0.097	0.028±0.024	0.250	-0.322±0.238	0.186
Playb.x act_{meas}	-0.003±0.011	0.805	-0.291±0.156	0.068	0.008±0.015	0.602	-0.115±0.182	0.532

Table S7: Outputs of the full linear mixed-effects models on metabolic rate (MR), evaporative water loss (EWL), relative water economy (RWE) and T_b of calm birds (i.e. $activity_{meas} \leq 3$) at the maximal T_a reached (i.e. T_a=42°C or 44°C, n=32 observations for 19 birds), within the thermoneutral zone (T_a=35°C, n=67 for 34 birds) and at mild temperature (T_a=27°C, n=677 for 34 birds). The reference is the control group for playback, morning for time-of-day (here "Time"), trial 1 for trial, and female for sex. Est. ± SE corresponds to estimate ± standard error. Bold font shows significant effect ($p<0.05$).

**EHL/MHP**		***Reduced model***			***Full model***		
	Predictors	*Est.*	*SE*	*p*	*Est.*	*SE*	*p*
**Above inflection point***	Intercept	0.923	0.045	**<0.001**	0.925	0.045	**<0.001**
	Playback	-0.013	0.035	0.714	-0.014	0.035	0.693
	$Mass_{init}$	0.007	0.015	0.653	0.008	0.016	0.650
	Time	-0.058	0.019	**0.006**	-0.059	0.019	**0.005**
	Trial	-0.036	0.020	0.081	-0.037	0.020	0.082
	Sex	0.057	0.035	0.117	0.056	0.035	0.124
	T_a	0.340	0.012	**<0.001**	0.350	0.014	**<0.001**
	$Activity_{meas}$	-0.044	0.012	**<0.001**	-0.044	0.012	**0.001**
	Playb. x T_a				-0.022	0.017	0.199
**Max T_a (42°C or 44°C)**	Intercept	1.385	0.182	**<0.001**	1.366	0.186	**<0.001**
	Playback	-0.104	0.069	0.164	-0.071	0.086	0.415
	$Mass_{init}$	-0.053	0.034	0.145	-0.052	0.034	0.152
	Time	-0.066	0.063	0.326	-0.029	0.085	0.743
	Trial	-0.183	0.073	**0.025**	-0.179	0.074	**0.034**
	$Activity_{meas}$	-0.054	0.044	0.231	-0.060	0.045	0.201
	Sex	0.001	0.072	0.990	-0.005	0.074	0.946
	T_a	0.244	0.109	**0.047**	0.241	0.110	0.052
	Playb. x time				-0.085	0.129	0.530
	Playb.x act_{meas}	-0.160	0.070	**0.037**	-0.158	0.071	**0.042**
**T_a=35°C**	Intercept	0.422	0.053	**<0.001**	0.419	0.053	**<0.001**
	Playback	0.002	0.042	0.962	0.019	0.048	0.696
	$Mass_{init}$	0.019	0.020	0.330	0.020	0.020	0.316
	Time	-0.066	0.022	**0.007**	-0.053	0.030	0.088
	Trial	-0.008	0.023	0.742	-0.010	0.023	0.670
	$Activity_{meas}$	0.006	0.015	0.699	-0.008	0.020	0.680
	Sex	0.105	0.042	**0.019**	0.104	0.042	**0.020**
	Playb. x time				-0.032	0.044	0.472
	Playb.x act_{meas}				0.035	0.030	0.256
**T_a=27°C**	Intercept	0.346	0.033	**<0.001**	0.338	0.032	**<0.001**
	Playback	-0.055	0.021	**0.016**	-0.038	0.025	0.138
	$Mass_{init}$	-0.003	0.010	0.777	-0.001	0.010	0.941
	Time	-0.018	0.013	0.169	<-0.001	0.016	0.989
	Trial	-0.036	0.017	**0.035**	-0.39	0.016	**0.020**
	$Activity_{meas}$	-0.002	0.010	0.803	0.005	0.011	0.663
	Sex	0.037	0.021	0.093	0.040	0.021	0.075
	Playb. x time				-0.037	0.024	0.135
	Playb.x act_{meas}				-0.025	0.016	0.139

Table S8: Outputs of the reduced and full linear mixed-effects models on evaporative cooling efficiency (EHL/MHP) of calm birds above the inflection point* (n=217 observations for 34 birds), at the maximal T_a reached (i.e. T_a=42°C or 44°C, n=32 observations for 19 birds), within the thermoneutral zone (T_a=35°C, n=67 for 34 birds) and at mild temperature (T_a=27°C, n=677 for 34 birds). The reference is the control group for playback, morning for time-of-day (here "Time"), trial 1 for trial, and female for sex. Est. ± SE corresponds to estimate ± standard error. Bold font shows significant effect ($p<0.05$). * Evaporative cooling efficiency (EHL/MHP) did not show any inflection point as a function of T_a from 35ºC (Davies test, p=1).

1 Mariette, M. M. & Buchanan, K. L. Prenatal acoustic communication programs offspring for high posthatching temperatures in a songbird. *Science* **353**, 812-814 (2016).
2 Mariette, M. M., Cathaud, C., Chambon, R. & Vignal, C. Juvenile social experience affects pairing success at adulthood: congruence with the loser effect? *Proc. Royal Soc.* **280**, 20131514 (2013).
3 Lighton, J. R. *Measuring metabolic rates: a manual for scientists*. (Oxford University Press, 2008).
4 Withers, P. C., Cooper, C. E., Maloney, S. K., Bozinovic, F. & Cruz-Neto, A. P. *Ecological and environmental physiology of mammals*. Vol. 5 (Oxford University Press, 2016).
5 Tracy, C.R., Welch, W.R., Pinshow, B. & Porter, W.P. Properties of air: a manual for use in biophysical ecology. 4th Ed. *The University of Wisconsin Laboratory for Biophysical Ecology: Technical Report* (2010)
